# Supplementary material for: Stable Superhydrophobic Aluminum Surfaces Based on Laser-Fabricated Hierarchical Textures
Source: Materials (Basel). 2021 Jan 2;14(1):184. doi: 10.3390/ma14010184 (PMC7795392; doi:10.3390/ma14010184)
Supplement: Supplementary file 1 [file materials-14-00184-s001.pdf]

materials

MDPI

Supplementary Materials

Stable Superhydrophobic Aluminum Surfaces Based on Laser-Fabricated Hierarchical Textures

Stephan Milles [1,*], Johannes Dahms [1], Marcos Soldera [1,2], and Andrés F. Lasagni [1,3]

[1] Institut für Fertigungstechnik, Technische Universität Dresden, George-Bähr-Str. 3c, 01069 Dresden, Germany; dahmsjohannes@gmail.com (J.D.); marcos.soldera@mailbox.tu-dresden.de (M.S.); andres_fabian.lasagni@tu-dresden.de (A.F.L.)
[2] PROBIEN-CONICET, Dto. de Electrotecnia, Universidad Nacional del Comahue, 8300 Neuquén, Argentina
[3] Fraunhofer-Institut für Werkstoff- und Strahltechnik (IWS), Winterbergstr. 28, 01277 Dresden, Germany
* Correspondence: stephan.milles@tu-dresden.de

Citation: Milles, S.; Dahms, J.; Soldera, M.; Lasagn, A.F. Stable SuperhyDrophobic Aluminum Surfaces Based on Laser-Fabricated Hierarchical Textures. *Materials* **2020**, *13*, 184. https://doi.org/10.3390/ma14010184

Received: 4 December 2020
Accepted: 28 December 2020
Published: 2 January 2021

Publisher's Note: MDPI stays neutral with regard to jurisdictional claims in published maps and institutional affiliations.

Copyright: © 2020 by the authors. Submitted for possible open access publication under the terms and conditions of the Creative Commons Attribution (CC BY) license (http://creativecommons.org/licenses/by/4.0/).

Table S1 provides the process parameters (spatial period, laser fluence and number of pulses) and the resulting structure depths on the Al-samples.

Table S1. Structure depth of the laser-fabricated textures on Al and the corresponding process parameters

Processing Method	Spatial Period (µm)	Sample Label (-)	Laser Fluence (J/cm²)	Pulses (#)	Structure Depth (µm)
DLIP	1.7	$DLIP_{1.7µm\ shallow}$	0.36	1	0.6
	1.7	$DLIP_{1.7µm\ deep}$	0.56	10	1.7
	3.4	$DLIP_{3.4µm\ shallow}$	0.82	2	1.8
	3.4	$DLIP_{3.4µm\ deep}$	1.33	15	3.9
	4.8	$DLIP_{4.8µm\ shallow}$	2.01	3	3.2
	4.8	$DLIP_{4.8µm\ deep}$	1.58	20	5.3
DLW	60	DLW	6.56	20	30.1
DLW+DLIP	60 + 1.7	$DLW+DLIP_{1.7µm\ shallow}$	6.56 + 0.56	20 + 1	29.1
	60 + 1.7	$DLW+DLIP_{1.7µm\ deep}$	6.56 + 0.56	20 + 10	28.1
	60 + 3.4	$DLW+DLIP_{3.4µm\ shallow}$	6.56 + 0.82	20 + 3	27.9
	60 + 3.4	$DLW+DLIP_{3.4µm\ deep}$	6.56 + 1.33	20 + 10	28.0
	60 + 4.8	$DLW+DLIP_{4.8µm\ shallow}$	6.56 + 2.01	20 + 7	28.3
	60 + 4.8	$DLW+DLIP_{4.8µm\ deep}$	6.56 + 1.58	20 + 15	27.9

In order to predict the WCA according to the Wenzel- and Cassie–Baxter models, their individual roughness factors need to be calculated. Therefore, the laser-textured surfaces are modelled as depicted in Figure S1. The colored area in Figure S1 indicates the area of the maximum structure height, which is wetted in the Cassie–Baxter model. The geometric parameters of the single-scale and multi-scale structures were measured on the real topography using confocal microscopy.

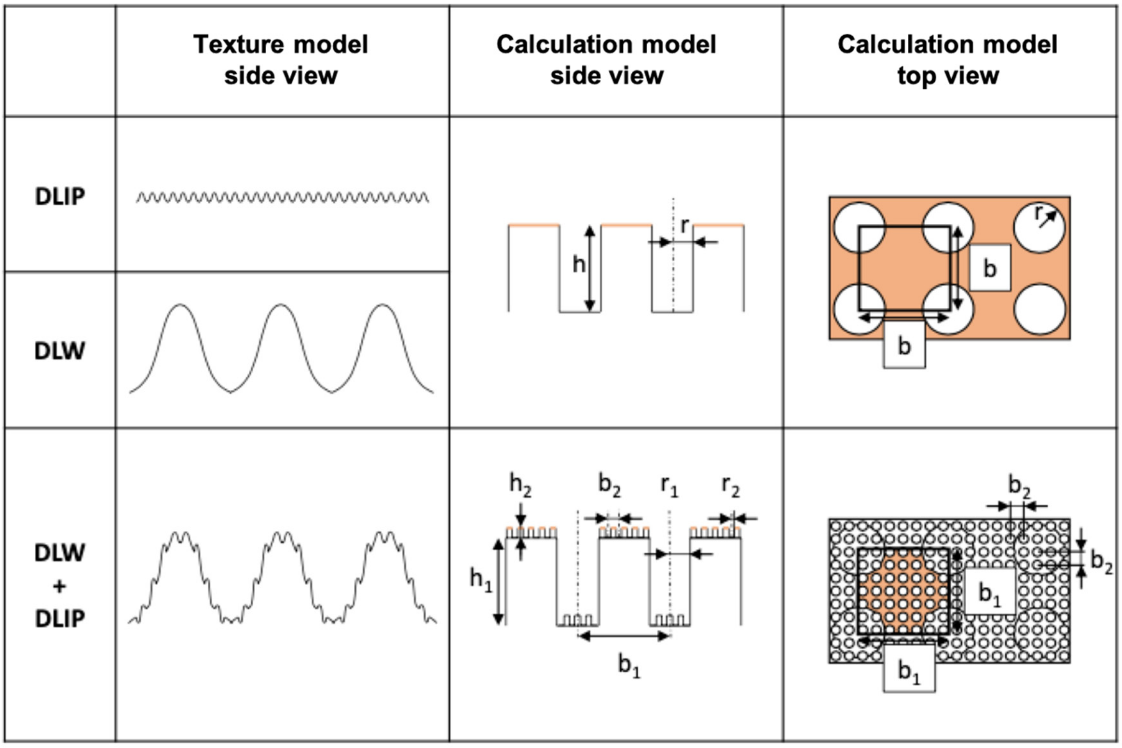

Figure S1. Overview of the calculation models applied for the single-scale and multi-scale textures.

According to Young's equation, a contact angle θ_Y of 95° was measured on an untreated reference surface. For the calculation of the static WCA of the single-scale structures, the following equations of the roughness parameters r_w for the wetting model according to Wenzel (Equation SE1) and f_{SL} for the wetting model according to Cassie–Baxter (Equation SE2) are given.

r_w defines the ratio of the real solid surface to the projected or apparent surface., while f_{SL} describes the ratio of the wetted projected area to the projected surface.

$$r_w = \frac{2 \cdot r \cdot h \cdot \pi + b^2}{b^2} \quad \text{(SE1)}$$

$$f_{SL} = \frac{b^2 - \pi \cdot r^2}{b^2} \quad \text{(SE2)}$$

where r is the radius of a DLW or DLIP shape element, h the structure height, and b the distance between two shape elements. In the following tables the corresponding structure parameters of the single-scale (Table S2) and of the multi-scale (Table S3) textures are shown according to Wenzel and Cassie–Baxter models.

Table S2. Model parameter for the single-scale textures based on confocal microscope analysis

Processing Method	Spatial Period (µm)	Sample Label (-)	Model Parameter (µm)			Roughness Parameter	
			h	r	b	r_W	f_{SL}
DLIP	1.7	DLIP$_{1.7µm\ shallow}$	0.6	0.5	1.7	1.67	0.72
	1.7	DLIP$_{1.7µm\ deep}$	1.7	0.5	1.7	2.85	0.73
	3.4	DLIP$_{3.4µm\ shallow}$	1.8	1.1	3.4	2.08	0.67
	3.4	DLIP$_{3.4µm\ deep}$	3.9	0.9	3.4	2.91	0.78
	4.8	DLIP$_{4.8µm\ shallow}$	3.2	1.8	4.8	2.57	0.56
	4.8	DLIP$_{4.8µm\ deep}$	5.3	1.7	4.8	3.46	0.61
DLW	60	DLW	30.1	25.0	60	2.31	0.45

Table S3. Model parameter for the multi-scale textures based on confocal microscope analysis

Processing Method	Spatial Period (µm)	Sample Label (-)	Model Parameter (µm)						Roughness Parameter	
			h_1	h_2	r_1	r_2	b_1	b_2	r_W	f_{SL}
DLW+DLIP	60 + 1.7	DLW+DLIP$_{1.7µm\ shallow}$	28.79	0.3	25	0.52	60	1.7	2.58	0.17
	60 + 1.7	DLW+DLIP$_{1.7µm\ deep}$	27.61	0.5	25	0.50	60	1.7	2.73	0.19
	60 + 3.4	DLW+DLIP$_{3.4µm\ shallow}$	27.12	0.8	25	1.10	60	3.4	2.63	0.15
	60 + 3.4	DLW+DLIP$_{3.4µm\ deep}$	26.92	1.1	25	0.90	60	3.4	2.68	0.25
	60 + 4.8	DLW+DLIP$_{4.8µm\ shallow}$	26.78	1.5	25	1.60	60	4.8	2.75	0.14
	60 + 4.8	DLW+DLIP$_{4.8µm\ deep}$	25.83	2.1	25	1.10	60	4.8	2.71	0.30
